# Fusion of Protegrin-1 and Plectasin to MAP30 Shows Significant Inhibition Activity against Dengue Virus Replication

**DOI:** 10.1371/journal.pone.0094561

**Published:** 2014-04-10

**Authors:** Hussin A. Rothan, Hirbod Bahrani, Zulqarnain Mohamed, Noorsaadah Abd Rahman, Rohana Yusof

**Affiliations:** 1 Department of Molecular Medicine, Faculty of Medicine, University of Malaya, Kuala Lumpur, Malaysia; 2 Genetics and Molecular Biology Unit, Institute of Biological Science, Faculty of Science, University of Malaya, Kuala Lumpur, Malaysia; 3 Department of Chemistry, Faculty of Science, University of Malaya, Kuala Lumpur, Malaysia; Temple University School of Medicine, United States of America

## Abstract

Dengue virus (DENV) broadly disseminates in tropical and sub-tropical countries and there are no vaccine or anti-dengue drugs available. DENV outbreaks cause serious economic burden due to infection complications that requires special medical care and hospitalization. This study presents a new strategy for inexpensive production of anti-DENV peptide-fusion protein to prevent and/or treat DENV infection. Antiviral cationic peptides protegrin-1 (PG1) and plectasin (PLSN) were fused with MAP30 protein to produce recombinant antiviral peptide-fusion protein (PG1-MAP30-PLSN) as inclusion bodies in *E. coli*. High yield production of PG1-MAP30-PLSN protein was achieved by solubilization of inclusion bodies in alkaline buffer followed by the application of appropriate refolding techniques. Antiviral PG1-MAP30-PLSN protein considerably inhibited DENV protease (NS2B-NS3pro) with half-maximal inhibitory concentration (IC_50_) 0.5±0.1 μM. The real-time proliferation assay (RTCA) and the end-point proliferation assay (MTT assay) showed that the maximal-nontoxic dose of the peptide-fusion protein against Vero cells is approximately 0.67±0.2 μM. The cell-based assays showed considerable inhibition of the peptide-fusion protein against binding and proliferating stages of DENV2 into the target cells. The peptide-fusion protein protected DENV2-challeged mice with 100% of survival at the dose of 50 mg/kg. In conclusion, producing recombinant antiviral peptide-fusion protein by combining short antiviral peptide with a central protein owning similar activity could be useful to minimize the overall cost of short peptide production and take advantage of its synergistic antiviral activities.

## Introduction

Dengue virus is a member of the *Flaviviridae* family, transmitted by the mosquito vector *Aedes aegypti* [1]. Dengue virus infects 50–100 million people worldwide each year and causes various clinical symptoms such as dengue fever (DF) that may later develop to severe dengue haemorrhagic fever (DHF), and dengue shock syndrome (DSS) [2]–[4]. Annually, there are approximately 0.5 million cases of DHF and DSS that lead to more than 20,000 deaths worldwide [5].The severe syndromes caused by dengue infection translate to serious economic burden in more than 100 tropical and sub-tropical countries [5]. Moreover, countries plagued with the dengue virus epidemic are mostly classified by World Bank as low income countries [6]. In view of that, there is an increased interest globally in developing new inexpensive vaccines and drugs as well as diagnostic tests that can be used for clinical management, and this would also be seen as a significant increase on the current support for new neglected tropical diseases technologies [7], [8].

Short cationic peptides have been considered as best drug leads for designing and developing new antiviral therapies [9]–[10]. The main reason for the interest in these peptides is that they possess high specificity and selectivity in their interactions and this ultimately reduce the possible side effects and maximize the potencies of action [11]. Previous studies reported significant inhibition of viral entry by synthetic peptides designed to target the last stage of virus fusion [12]. In addition, antimicrobial cationic peptides like Protegrin-1 (PG1) have been shown to inhibit the dengue NS2B-NS3pro that in turn impairs viral replication in the host cells [13]. One of the main hindrances for successful production of these peptides using chemical synthesis are the high costs involved, and is currently deemed uneconomic especially to achieve the required volumes for epidemic response. As an alternative, production of these peptides in recombinant form would be cost-effective if a suitable expression system is establish to be scalable and well suited for mass production of bioactive peptides.

*Escherichia coli* and its various strains have been widely used as economic expression systems to produce numerous foreign proteins. Unfortunately, there are considerable limitations in using this system to produce bioactive antiviral peptides. One of these limitations is the low efficiency in the formation of disulphide bonds for cysteine-rich peptides that is important for antimicrobial bioactivity [14]. In addition, short peptides are almost always produced in soluble form and are often misfolded. This necessitates additional steps like in-column refolding and purification, and thus represents a considerable problem to large-scale production efforts [15]. Producing antiviral peptides in *E. coli* as inclusion bodies could represent an attractive solution to the problem above, and to facilitate high yield production. This approach requires only a few washing steps to isolate the inclusion bodies, and this is then followed by the appropriate refolding technique [16], [17]. Our previous work reported production of the plectasin peptide in inclusion bodies by tandem fusion of two peptide units separated by a protease recognition site [16]. This strategy required extra steps of enzyme digestion and elimination of enzyme residues from the final products. The current study presents a new approach in which functional recombinant cationic peptides are produced as parts of a peptide-fusion protein. This protein was designed to harbour antiviral peptides fused to a central antiviral protein. The central protein MAP30, an antiviral protein isolated and purified from the fruit and seeds of the Momordica charantia (or commonly known as bitter gourd, has been previously shown to be successfully produced in *E. coli* as inclusion bodies [18].

In this study, the short cationic peptides protegrin-1 (PG1) and plectasin (PLSN) were doubly fused with a central protein, MAP30, to produce a recombinant antiviral peptide-fusion protein (PG1-MAP30-PLSN). PG1 is originally isolated from porcine white blood cells and has been considered as a potent antibiotic agent against a broad range of microorganisms [19], [20]. PLSN, on the other hand is the first antimicrobial fungus-derived defensin, produced by the fungus *Pseudoplectania nigrella* with secondary structures similar to those of defensins found in other organisms [10], [21]. These two peptides PG1 and PLSN are fused to MAP30 as an anchoring central antiviral protein. MAP30 is a 30 kDa type-I ribosome inactivating protein (RIP) possessing anti-HIV activities [22], [23]. In terms of their antiviral activity, both PG1 and PLSN have been previously shown to possess considerable inhibition potential against dengue NS2B-NS3 serine protease and virus replication *in vitro* [13], [16]. There are currently no reports on whether or not MAP30 possesses an inhibition potential towards dengue virus. As such, this study was also designed to address this question.

## Methods

### Design of antiviral peptide-fusion protein

The peptide-fusion protein consisted of PG1 and PLSN as flanking peptides fused to MAP30 as a central protein. The peptide-fusion protein PG1-MAP30-PLSN protein was constructed by joining the C-terminal of PG1 with the N-terminal of MAP30 by using a ten amino-acid linker, and the C-terminal of MAP30 was joined to the N-terminal of PLSN by using another ten amino-acid linker.

### Production of recombinant proteins in *E. coli*

**Figure 1:**
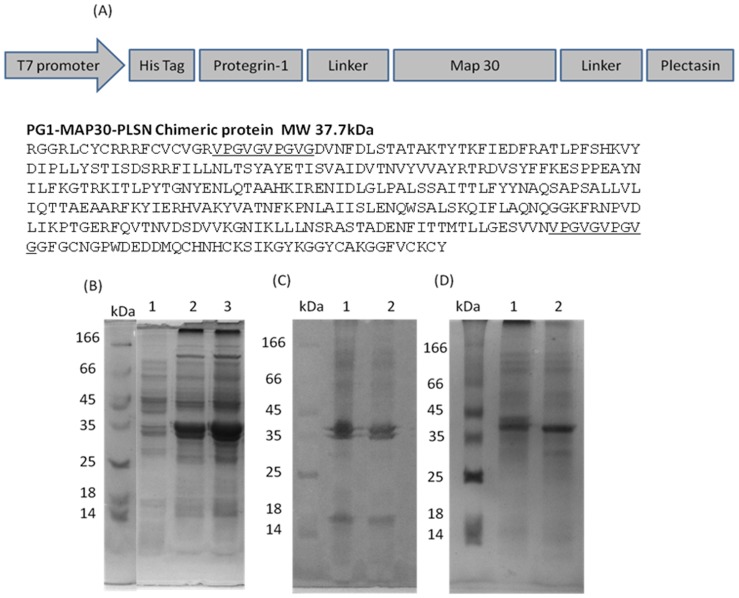
Production of recombinant peptide-fusion protein (PG1-MAP30-PLSN) in *E. coli* as inclusion bodies. (A) Design of peptide-fusion protein: PG1 peptide was joined with N-terminal of MAP30 by 10-amino-acid linkers (underlined) and PLSN peptide was joined to the C-terminal of MAP30 by similar linkers. (B) The peptide-fusion protein was produced insolubly as inclusion bodies: Lane 1, before induction with IPTG; Lane 2, expression of peptide-fusion protein after induction; Lane 3, expression of MAP30 after induction. (C) Isolation of inclusion bodies by multiple washing steps: Lane 1, peptide-fusion protein; Lane 2, MAP30. (D) Inclusion bodies were solubilized and refolded in an alkaline buffer containing redox agents: Lane 1, peptide-fusion protein; Lane 2, MAP30.

The recombinant peptide-fusion protein (PG1-MAP30-PLSN) was produced in *E. coli*. For comparison purposes, the PLSN peptide and MAP30 were produce in recombinant form as previously described [16], [18] while PG1 was chemically manufactured by standard solid-phase peptide synthesis [13]. The DNA sequence of the peptide-fusion protein (*PG1-MAP30-PLSN*) (Fig.1A) was obtained by reverse translating the amino acid sequence and optimized to *E. coli* preferred codons as previously describe [24], [25] using software available online. Alternating sense and antisense oligos of 60-mers in length (with 15 bp overlap region) were designed to span the entire PG1-MAP30-PLSN expression cassette and synthesized commercially (1^st^base, Kuala Lumpur–Malaysia) (Data S1). Splicing and synthesis of the entire PG1-MAP30-PLSN expression cassette was achieved using Klenow-*Pfu* DNA polymerase method [26]. The PG1-MAP30-PLSN expression cassette (and the individual MAP30 gene) was amplified using forward and reverse primer that were designed to include *Bam*HI and *Hind*III restriction sites respectively. Then, the PG1-MAP30-PLSN expression cassette or MAP30 was digested with *BamHI* and *Hind*III enzymes to facilitate cloning into an appropriate *E. coli* expression vector (pTrc-His-A, Invitrogen, Cat. no. V360-20). To isolate inclusion bodies, bacterial cells were harvested and lysed by sonication in lysis buffer. Following a centrifugation step, the isolated inclusion bodies were subjected to excessive washing steps and solubilized by NaOH. This was then followed by protein refolding steps as described previously [27]. Further purification was carried out using column chromatography to eliminate host cell contamination from the final product.

### Dengue NS2B-NS3 protease (NS2B-NS3pro) assay

The assay was carried out to examine the ability of antiviral peptides to inhibit DENV2 dengue serine protease (NS2B-NS3pro) [13, 15 and 16]. In brief, a single chain NS2B (G_4_-T-G_4_) NS3pro was produce as a recombinant protein in *E. coli* [28], [29]. The end point reaction mixture was performed in black 96-well plates which contained 2 μM recombinant NS2B-NS3pro, 100 μM fluorogenic peptide substrate (Boc-Gly-Arg-Arg-AMC) and antiviral peptides of varying concentrations, buffered at pH 8.5 with 200 mM Tris-HCl with total volume of 200 μl. The reaction mixture without antiviral peptides, substrate with antiviral peptides, enzyme and different concentrations of antiviral peptides were used as controls. Thereafter, all reaction mixtures were incubated at 37°C for 30 minutes and the substrate was added to the specific reaction mixtures and incubated at the same temperatures for another 30 minutes. Measurements were performed in triplicates using Tecan Infinite M200 Pro fluorescence spectrophotometer (Tecan Group Ltd., Switzerland). Substrate cleavage was normalized against buffer only (control) at the emission of 440 nm upon excitation at 350 nm. The IC_50_ was calculated from nonlinear regression fitting of signal *vs.* concentration data points to the standard dose–response equation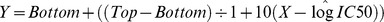. In this equation, *X* was the log of compound concentration, *Y* was the response signal, and bottom and top refer to plateaus of the sigmoid response curve. All assays were performed in triplicate and repeated twice. The inhibition percentage was calculated using the following formula: 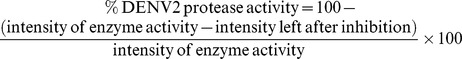


### Maximum non-toxic dose test (MNTD)

The MNTD assay was carried out to determine the maximal concentration with lessen cytotoxic effects of the antiviral peptides. The MNTD test was initiated by seeding Vero cells at 1×10^4^ cells/well in triplicates, at optimal conditions (37°C, 5% CO_2_ in humidified incubator) in 96 well plates with blank control (media only) and cells control (cells only). After overnight incubation, the cells were treated with increased concentrations of each antiviral peptide with DMEM media supplemented with 2% FBS. The cell culture was analyzed after 72 h using Non-Radioactive Cell Proliferation assay (Promega, USA) according to the manufacturer's protocol. The MNTD was calculated from dose-response curves and the percentage of cell viability was calculated as follows: 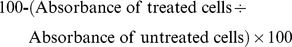



### Real-Time Cell Proliferation Assay (RTCA assay)

This assay was carried out to test the real time effects of the peptide-fusion protein on cell viability. Cell proliferation was measured using xCELLigence Real-Time Cellular Analysis (RTCA) system (Roche, Germany) as described previously [30]. Cell viability and growth was monitored continuously after applying increased concentrations of PG1-MAP30-PLSN protein. Briefly, background measurements were taken after adding 100 μl of the culture medium to the wells. Next, cells were seeded at a density of 1×10^4^ cell/well on a 16-well plate with electrodes for 18 h to allow cells to grow to the log phase. Cells were treated with different concentrations of the compounds dissolved in cell culture media and continuously monitored for up to 100 h. Cell sensor impedance was expressed as an arbitrary unit called the Cell Index. Cell index were recorded every 5 min by RTCA analyzer. To eliminate variation between wells, cell index values were normalized to the value at the beginning of treatment time-point.

### Treatment of DENV2-infected cells with antiviral peptides

To infect Vero cell lines with dengue virus (DENV2-isolate Malaysia M2, GenBank Toxonomy No.: 11062), the cells were cultured in 24-well plates (1.5×10^5^ cells/well) for 24 h at 37°C and 5% CO_2_. Virus supernatant was added to the cells at multiplicity of infection (MOI) of 0.2 followed by incubation for 1 h with gentle shaking every 15 min for optimal virus to cell contact. The cells were washed twice with fresh serum-free DMEM media after removing the virus supernatant. Then, new complete DMEM media mixed with each peptide were separately added and the cultures were incubated for 24, 48 and 72 h. Afterwards, cellular supernatants were collected and stored at −80°C for viral load quantification.

### Virus binding assay

This test was carried out to examine the ability of the peptides in inhibiting virus binding to the host cells. Vero cells were grown in six-well microplates (1.5×10^6^ cells/well) for 24 h. Cell culture media were removed and the cells were washed three times with PBS. Then, new media containing virus supernatant mixed with each peptide were separately added and the cells were incubated for 1 h at 4°C. The media were removed and the cells were washed extensively with cold PBS to remove the unadsorbed virus. Cells were harvested at 24, 48 and 72 h and the viral RNA was quantified by qReal time-PCR.

### Virus quantification by plaque formation assay

To determine the virus yield after treatment with different concentrations of the peptides, culture supernatants were collected and serially diluted to reduce the effects of drug residues. A 10-fold serial dilution of medium supernatant was added to new Vero cells grown in 24-well plate (1.5×10^5^ cells) and incubated for 1 h at 37°C. The cells were then overlaid with DMEM medium containing 1.1% methylcellulose. Viral plaques were stained with crystal violet dye after five-day incubation. Virus titers were calculated according to the following formula: Titer (p.f.u./ml)  =  number of plaques/volume of diluted virus added to the well × dilution factor of the virus used to infect the well in which plaques were enumerated.

### Quantitative real-time PCR

After treatment with antiviral peptides, the RNA copies of DENV2 were quantified using One-step Real-time PCR as previously described [13]. In brief, a standard curve was generated by 10-fold serial dilution of known copies of DENV2 RNA. Then, viral RNA was extracted from culture supernatant using QIAmp viral RNA extraction kit (QIAGEN, Germany). The qRT-PCR was carried out using SyBr Green Master Kit (Qiagen, Germany) in quadruple experiments and absolute quantification was performed using ABI7500 machine (Applied Biosystems, Foster City, CA). Results were analyzed using Sequence Detection Software Version 1.3 (Applied Biosystems, Foster City, CA).

### Indirect immunostaining

In order to examine the uptake of the peptide-fusion protein by Vero cells, the cells were grew on the cover slide in 6 well plates and treated with the peptide-fusion protein for 24 h. Then, the cells were washed three times with PBS to remove the residues of the peptide and fixed with ice-cold methanol for 15 min at −20°C. After washing steps, the cells were incubated with coating buffer for 1 h at room temperature. Mouse anti-His tag antibody was added and the cells were incubated overnight at 4°C. The cells were washed three times with PBS and incubated for 30 min with anti-mouse IGg labelled with FITC fluorescence dye and the Hoechst dye was added at the last 15 min of the incubation period.

### Animal experiments

ICR mice were handled in accordance with University of Malaya guidelines on the care and use of laboratory animals. The Animal Ethics Committee of the University of Malaya approved all experimental procedures that were used in the present study. Mice were used at 3 to 5 weeks of age and average body weight of approximately 30 g. The animals were firstly used to evaluate the lethal dose of the peptide- fusion protein that kills 50 percent of the animals (LD_50_). Two groups of animals (n = 12 each, 6 females and 6 males) were intraperitoneally administrated with 5 mg/kg as a low dose (0.25 ml of 0.6 mg/ml of the peptide-fusion protein) and 50 mg/kg as a high dose (0.25 ml of 6 mg/ml of the peptide-fusion protein). The animals were observed for 24 h without any signs of toxicity, after 14 days post- treatment, the animals were healthy and no signs of toxicity or death cases were recorded. Then, four groups of animals (n = 6 each) were intraperitoneally inoculated with 4×10^3^ plaque-forming units (PFU) of the purified DENV2 (DENV2-isolate Malaysia M2, GenBank Toxonomy No.: 11062). Simultaneously, three groups were individually administrated with 12.5, 25 and 50 mg/kg of the peptide-fusion protein by intraperitoneal administration; while the forth group was administrated with PBS as a mock-administrated group. Mice were observed for 7 days post-infection and the death cases were recorded and Kaplan-Meier analysis was used to generate survival curves with Prism software 5.01 (GraphPad Software, San Diego, CA).

### Statistical analysis

All the assays were done in triplicates and the statistical analyses were performed using GraphPad Prism version 5.01 (GraphPad Software, San Diego, CA). *P* values of <0.05 were considered significant. Error bars are expressed as ± SD.

## Results

### Production of recombinant peptide-fusion protein (PG1-MAP30-PLSN)

PG1 peptide was joined to the N-terminal portion of MAP30 by using a 10-amino-acid linker and PLSN peptide was joined to the C-terminal of MAP30 using a similar linker. The DNA sequence coding the peptide-fusion protein expression cassette (PG1-MAP30-PLSN) was optimized for *E. coli* preferred codon and constructed *in vitro* using Klenow-*Pfu* DNA polymerase procedure. The molecular weight of the resulting peptide-fusion protein was approximately 37.7 kDa (Fig. 1A). The peptide-fusion protein was produced insolubly as inclusion bodies at high levels reaching approximately 80% of the total bacteria protein (Fig. 1B). Inclusion bodies were isolated by washing steps to eliminate host cell proteins (Fig. 1C). In order to retrieve the bioactivity of recombinant peptide-fusion protein, isolated inclusion bodies was solubilized and refolded in alkaline-based buffer containing redox agents (Fig. 1D). The recombinant peptide-fusion protein was designed to include 6X His-tag to facilitate purification by Ni-affinity chromatography. The antimicrobial activity of the recombinant protein was confirmed as it showed high inhibition potential against gram-positive and gram-negative bacteria (data not shown).The total amount of recombinant peptide-fusion protein was approximately 30 mg per litre of *E. coli* cell culture.

### Inhibition of dengue protease by antiviral peptide-fusion protein

**Figure 2:**
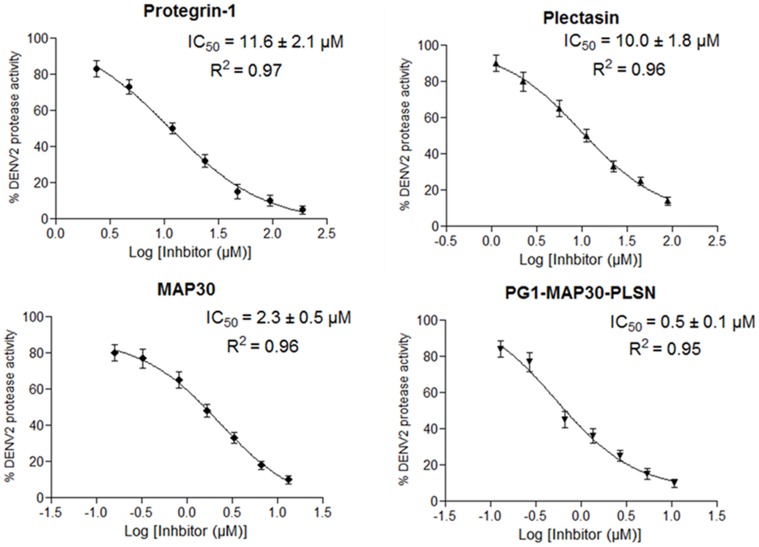
Evaluation of the inhibitory effects of the peptide-fusion protein and the individual antiviral peptides against dengue NS2B-NS3 protease. Recombinant dengue NS2B-NS3pro was produce in *E. coli* and used in an end point reaction mixture with fluorogenic peptide substrate, antiviral peptides of varying concentrations and buffer. The antiviral peptides showed significant dose-dependent inhibition towards dengue NS2B-NS3pro. The peptide-fusion protein inhibited dengue protease at IC_50_ value lower than MAP30, PG1 and PLSN.

Dengue NS2B-NS3 protease (NS2B-NS3pro) assay was carried out to evaluate the inhibition potential of the peptides individually or as peptide-fusion protein. The results showed that all peptides exhibited significant dose-dependent inhibition against dengue NS2B-NS3pro (Fig. 2). The peptide-fusion protein showed significantly higher inhibition potential against dengue NS2B-NS3pro (IC_50_, 0.50±0.1 μM) compared to individual MAP30 (IC_50_, 2.3±0.5 μM), PG1 (IC_50_, 11.6±2.1 μM) and PLSN (IC_50_, 10.0±1.8 μM) (Fig. 2).

### Evaluation of peptide cytotoxicity

**Figure 3:**
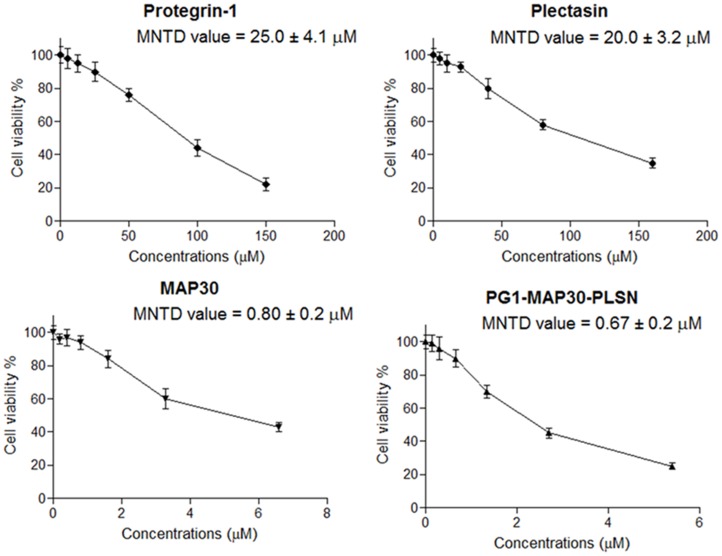
Evaluation of peptide cytotoxicity. Toxicity was measured to determine the maximal non-toxic dose test (MNTD) value of the inhibitory peptides, which is the highest concentration of the peptide that causes minimal toxic effects on the cells. This assay was carried out by seeding Vero cells at 1×10^4^ cells/well in triplicates and treated with increased concentrations of antiviral peptides. The cell culture was analyzed after 72 h using Non-Radioactive Cell Proliferation assay. In this study, the peptide concentration that showed 90% and above of the cell viability was considered as the MNTD value, assuming that approximately 90% of the cells were healthy.

Toxicity was measured to determine the maximum non-toxic dose (MNTD) of the inhibitory peptides. Besides the undesired effect, toxicity could induce cellular alterations that decrease the formation of plaques leading to false interpretation of antiviral activity. Toxic effects ranged from no evidence to minimal toxicity for different peptides when compared to untreated control cells. In order to evaluate the anti-dengue properties of the peptide-fusion protein in comparison to the peptides acting individually, all peptides were first subjected to a toxicity test. In this study, the MNTD values were determined using serially diluted peptides followed by further optimization in order to achieve a specific cytotoxic concentration. The MNTD value of each peptide obtained through the optimization steps are presented in Figure 3. The MNTD value of PG1 (25.0±4.1 μM), PLSN (20.0±3.2 μM), MAP30 (0.80±0.2 μM) and PG1-MAP30-PLSN (0.67±0.2 μM) were used in the following experiments.

### Cellular uptake of the peptide-fusion protein and real-time cell proliferation assay

**Figure 4:**
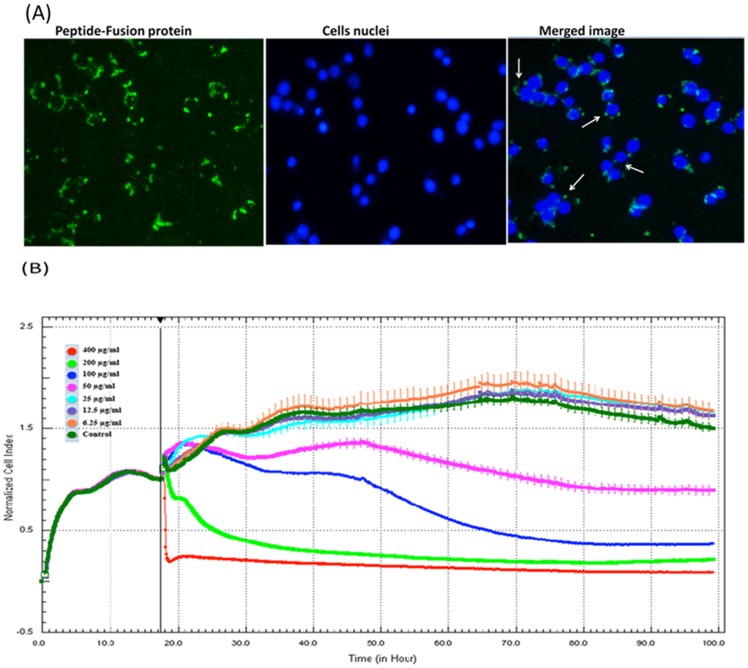
Peptide-fusion protein uptake and real time cell proliferation assay. (A) Cellular uptake of the peptide-fusion protein was analyzed by immunostaining images that show localization of peptide-fusion protein around cells nuclei. (B) Cell proliferation was measured using xCELLigence Real-Time Cellular Analysis (RTCA) system. Cell viability and growth was monitored continuously after incubating the cells for 18 h at standard condition and applying increased concentrations of the PG1-MAP30-PLSN protein (6.25, 12.5, 25, 50, 100, 200 and 400 μg/ml or 0.17, 0.34, 0.67, 1.35, 2.70, 5.40 and 10.81 μM).The effects of the peptide-fusion protein on cell proliferation were insignificant at the doses less than 25 μg/ml (0.67 μM) for 82 h as the cell index was similar with untreated control cells. Cell proliferation was considerably decreased at the dose more than 50 μg/ml (1.35 μM).

To evaluate the internalization of the peptide-fusion protein into Vero cells, the intracellular PG1-MAP30-PLSN protein was targeted by anti-His tag antibody and secondary antibody conjugated with FITC. The result showed that the targeted protein was distributed around the cells nuclei (Fig. 4A). As mentioned earlier, our antiviral peptide-fusion protein (PG1-MAP30-PLSN) contains MAP30 as a central protein, which possesses ribosome-inactivation activities [23]. This activity of MAP30 could induce cellular alterations that decrease the formation of plaques leading to false interpretation of antiviral activity. To clarify this issue, we examined the effects of increased concentrations of the peptide-fusion protein on real time cell proliferation using the Real-Time Cellular Analysis (RTCA) system. The cells were incubated for 20 h without treatment and then treated with increased concentration of the peptide-fusion protein. The results showed that the effects of peptide-fusion protein on cell proliferation were insignificant at the dose of 25 μg/ml (0.67 μM) for 82 h as the cell index was similar with untreated control cells. Cell proliferation was considerably decreased at the dose of more than 50 μg/ml (1.35 μM) (Fig. 4).

### Assessment of antiviral activity

**Figure 5:**
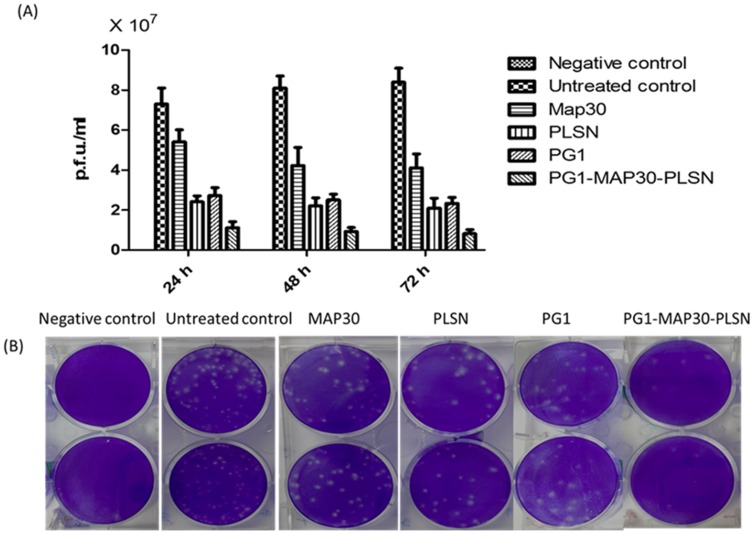
Evaluation of the peptides antiviral activities using plaque formation assay. (A) Virus load expressed as plaque forming units per ml (p.f.u/ml) was significantly reduced after treatment with all the peptides compared to untreated cells. The peptide-fusion protein (PG1-MAP30-PLSN) showed the highest inhibition potential compared with the other peptides. (B) Plaque formation assay shows the reduction of plaque generation after the treatment of the infected cells with the peptides. (Two-way ANOVA with Bonferroni post-test, p<0.001).

The effectiveness of the peptides was verified by testing the antiviral activity of the peptides against DENV2 using plaque formation assay and qRT-PCR analysis. The results showed significant (p<0.001) reduction in the DENV2 load that was expressed as plaque forming units per ml (p.f.u./ml) after treatment with all the peptides compared to untreated cells (Fig. 5A and 5B). The peptide-fusion protein showed the highest reduction in the viral load (1.21±0.3 ×10^7^, 0.93±0.2×10^7^ and 0.82±0.2×10^7^ p.f.u./ml at 24, 48 and 72 h respectively) compared with untreated control (7.40±0.8×10^7^, 8.10±0.6×10^7^ and 8.41±0.7×10^7^ at 24, 48 and 72 h respectively). While, MAP30 showed the lowest antiviral activity amongst the other peptides (5.41±0.6×10^7^, 4.23±0.9×10^7^, 4.11±0.7×10^7^ p.f.u./ml at 24, 48 and 72 h respectively). Interestingly, both of PG1-MAP30-PLSN and MAP30 showed significant (p<0.05) time-dependent antiviral activity. The inhibition potential of PG1 and PLSN was approximately similar, which was significantly (p<0.001) higher than MAP30 and lower than PG1-MAP30-PLSN with insignificant time-dependent effects.

**Figure 6:**
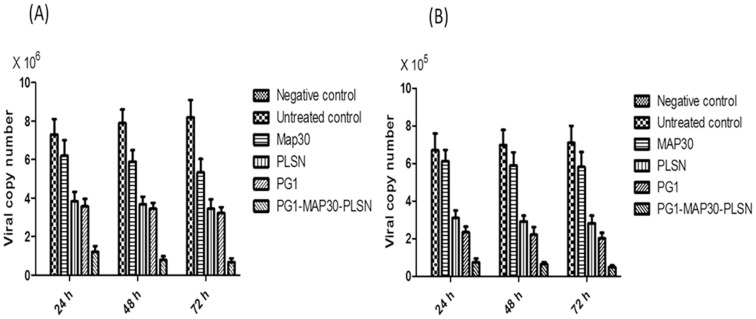
Evaluation of the peptides antiviral activities using qRT-PCR. Viral RNA levels were quantified by qRT-PCR to determine the level of viral copy number after the treatment of DENV2-infected cells with the antiviral peptides. (A) Viral copy number was reduced after the treatment with the peptides compared with untreated control. The peptide-fusion protein (PG1-MAP30-PLSN) showed the highest reduction in the viral copy number amongst the other peptides in a time-dependent manner. (B) Inhibition of virus binding to the target cells that led to reduce dengue virus copy number after the treatment with the peptides. The peptide-fusion protein potentially inhibited virus binding to the target cell by considerable reduction in the viral copy number compared with untreated control. However, there was insignificant (p>0.05) effect of MAP30 on virus binding to the target cell. PG1 and PLSN similarly inhibited virus binding at significant levels. Results are expressed as mean ± SD from a representative experiment performed in quadruple experiments. (Two-way ANOVA with Bonferroni post-test, p<0.001).

These results were further verified by quantification of viral copy number by evaluating viral RNA levels using qRT-PCR analysis. The results showed that the peptide-fusion protein was significantly (p<0.001) able to inhibit virus replication in Vero cells by reducing viral copy number (1.20±0.3×10^6^, 0.78±0.2×10^6^ and 0.67±0.2×10^6^ at 24, 48 and 72 h respectively) compared with untreated control (7.30±0.9×10^6^, 7.91±0.7×10^6^ and 8.22±0.6×10^6^ at 24, 48 and72 h). In addition, MAP30 showed the lowest inhibitory effect (p<0.001) amongst the other peptides (5.41±0.6×10^6^, 4.23±0.9×10^6^ and 4.11±0.7×10^6^ at 24, 48 and 72 h respectively). In parallel with the results of plaque formation assay, both PG1-MAP30-PLSN and MAP30 showed time- dependent inhibitory effects. However, PG1 and PLSN showed insignificant time-dependent effects. Both of PG1 and PLSN showed similar inhibition activities that were lower than PG1-MAP30-PLSN and higher than MAP30 (Fig. 6A).

The results also showed significant (p<0.001) reduction in viral copy number when DENV2 was individually incubated with each peptide to examine the effect of the peptides on virus binding to the target cell. The peptide-fusion protein (PG1-MAP30-PLSN) potentially inhibited virus binding to the target cell as evidenced by a considerable reduction in the viral copy numbers (0.74±0.2×10^5^, 0.65±0.1×10^5^ and 049±0.1×10^5^ compared with untreated control 6.70±0.9×10^5^, 6.98±0.8×10^5^ and 7.10±0.9×10^5^ at 24, 48 and 72 h respectively). However, there was insignificant (p>0.05) effect of MAP30 on virus binding to the target cell while PG1 and PLSN significantly (p<0.001) inhibited virus binding to the target cell as presented in Figure 6A and 6B.

### Determination of the antiviral peptide-fusion protein effective dose

**Figure 7:**
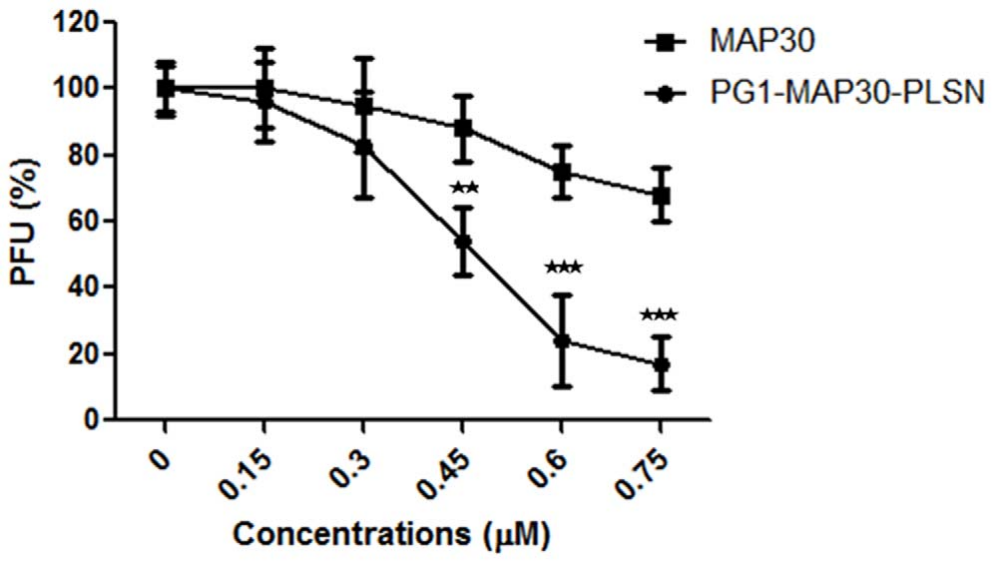
Dose-response curves for peptide-fusion protein and MAP30. The figure shows the effect of increasing concentrations of the peptide-fusion protein and MAP30 peptides against DENV2. The peptide-fusion protein showed a maximum inhibitory activity against DENV2 of 18.6%±7.2 at 0.75 μM with EC_50_ about 0.43 μM. MAP30 showed a maximum inhibition activity against DENV2 of 68.9%±6.7 at 0.75 μM with EC_50_ more than 0.75 μM. Results are expressed as mean ± SD from a representative experiment performed in triplicate. Asterisk denotes statistically significant differences between peptide-fusion protein and MAP30. (Two-way ANOVA with Bonferroni post-test, p<0.001).

Dose response curves were generated for the recombinant peptide-fusion protein in comparison with MAP30 against DENV2 to determine the effective dose that inhibits 50% of the viral activity (EC_50_). The inhibitory activity increased with increasing concentrations of both recombinant proteins. Interestingly, the peptide-fusion protein showed a maximum inhibition activity against DENV2 of 18.6%±7.2 at 0.75 μM with EC_50_ about 0.43 μM, while MAP30 showed a maximum inhibition activity against DENV2 of 68.9%±6.7 at 0.75 μM with EC_50_ more than 0.75 μM as shown in Figure 7.

### Protection against DENV2 infection

**Figure 8:**
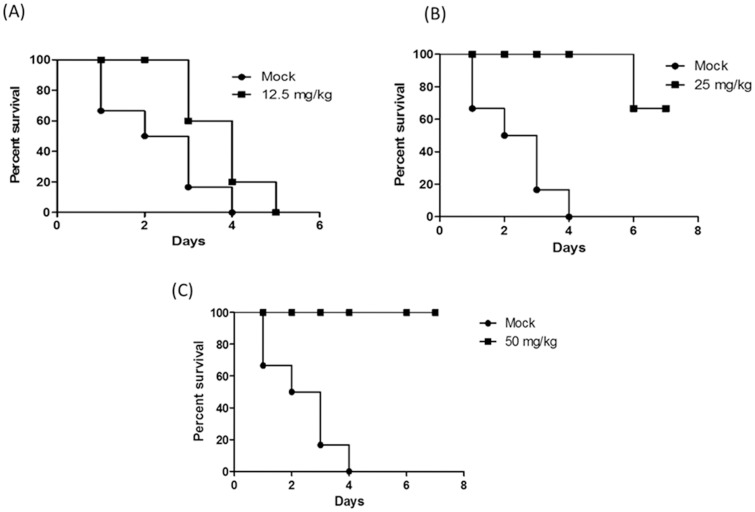
The percentage of survival of DENV2-challenged mice administered with peptide-fusion protein. The lethal dose of the peptide- fusion protein that kills 50 percent of the animals (LD_50_) was evaluated to be more than 50 mg/kg. Then, four groups of the animals (n = 6) were inoculated with 4×10^3^ plaque-forming units (PFU) of the purified DENV2 (DENV2-isolate Malaysia M2, GenBank Toxonomy No.: 11062) and treated with 12.5, 25 and 50 mg/kg of the peptide-fusion protein by intraperitoneal administration; while the forth group was treated with PBS as a mock-administrated group. (A), (B) and (C) The survival percentage of the animals that were administrated with 12.5, 25 and 50 mg/kg of the peptide-fusion protein respectively, compared with the mock-administrated group. Kaplan- Meier analysis was performed to generate survival curves with Prism software 5.01 (GraphPad Software, San Diego, CA).

The ICR mice were intraperitoneally administrated with low dose (5 mg/kg) and high dose (50 mg/kg) of the peptide-fusion protein. The animals were kept for 14 days post-administration with no signs of toxicity or death cases were observed. Therefore, we assumed that the LD_50_ value of the peptide- fusion protein could be more than 50 mg/kg. Based on that, the doses that were used in the following experiment were equal or less than 50 mg/kg to eliminate the possible cytotoxic effect of the peptide-fusion protein that may cause animal death. DENV2-chalenged animals were simultaneously treated with 12.5, 25 and 50 mg/kg of the peptide fusion protein and kept for 7 days post-infection. The results showed that the peptide-fusion protein protected DENV2-challenged animals in a dose-dependent manner. All the animals of the mock-administrated group were died at the period of 4 days post-infection and 5 of 6 animals that administrated with 12.5 mg/kg of the peptide- fusion protein were died at the period of 5 days post-infection (Fig. 8A). However, only one animal of the group that was administrated with 25 mg/kg was died at the seventh day of the experiment (Fig. 8B). Interestingly, the peptide-fusion protein showed 100% of survival at the dose of 50 mg/kg as presented in Figure 8C.

## Discussion

Our previous studies have shown that PG1 and PLSN exhibited significant inhibition against dengue serine protease (NS2B-NS3pro) and dengue replication in infected cells. We then hypothesized that the design and construction of a recombinant antiviral peptide-fusion protein by fusing these peptides to a central antiviral protein will provide two significant advantages over conventional approaches. Firstly, the recombinant peptide-fusion protein can synergistically result in amplified antiviral activity. Secondly, the use of MAP30, a protein already known to be successfully expressed through inclusion bodies, as the anchoring central protein would facilitate high yield production in *E. coli.*

Producing antiviral peptides in inactive and insoluble (inclusion bodies) form by *E. coli* has considerable advantages over active and soluble form. Producing recombinant protein in inclusion bodies is considered a scalable strategy for recombinant protein production which has been applied for the production of high quantities of recombinant globular adiponectin [17]. Furthermore, formation of inclusion bodies is also important to protect the host cells from the toxic effects of the expressed product. This strategy was useful in producing recombinant phage T4 restriction endoribonuclease which is highly toxic to the host cells [31]. As such, antiviral activity of the peptide-fusion protein was retrieved after solubilization of inclusion bodies in alkaline buffer followed by refolding of the soluble protein in buffer containing redox agents. It is important to note that the activity of antiviral peptides almost always depends on its secondary structure which is maintained by the formation of intra-molecule disulphide bonds [32]. Therefore, reformation of disulphide bones was achieved in this study by reducing the existence bonds and reforming it in the presence of reduced and oxidized glutathione as previously reported [27].

The results of this study showed that the peptide-fusion protein (PG1-MAP30-PLSN) as well as, to a lesser extent, its individual components significantly inhibited dengue NS2B-NS3pro. Inhibition of dengue NS2B-NS3pro has been considered as a target to develop anti-dengue drugs [4]. It is now known that the post-translational proteolytic cleavage of viral precursor protein is largely dependent on dengue NS2B-NS3pro and host cell proteases. This process results in the formation of three structural proteins and seven non-structural viral proteins that is later required for dengue virus propagation [33]–[34]. Therefore, inhibition of dengue NS2B-NS3pro leads to impaired production of viral proteins which eventually leads to reduced virus replication capabilities. Importantly, the binding between dengue protease subunits depends on the interaction between negatively charged amino acids of NS2B and positively charged amino acids of NS3 [35]. The possible action of cationic peptides like PG1 and PLSN is to interrupt the binding of NS3 with its co-factor that leads to reduce enzyme activity. Other peptides that contain α-helical structure similar to the PLSN peptide have shown considerable inhibitory effects against HIV-1 due to an actual interference with the virus assembly stage in the viral life cycle [36]. Similarly, the PG1 peptide that possess a strong positive charge, have shown considerable antiviral activity against the dengue virus protease that is important for post-translational processes [13]. Therefore, the antiviral activity of the PLSN and PG1 peptides against dengue is proposed to be due to their interference with the viral replication stages into the host cells. Consequently, the inhibition potential of peptide-fusion protein (PG1-MAP30-PLSN) against dengue NS2B-NS3pro might be one of the main causes of viral reduction in cell culture after treatment with peptide-fusion protein.

The current study showed considerable inhibition of MAP30 and higher inhibition potential of peptide-fusion protein (PG1-MAP30-PLSN) against viral replication. Besides, the data of this study also confirmed our previous findings of the inhibition potential of PG1, PLSN against dengue virus replication [13], [16]. It has been shown that these peptides exhibited similar antiviral activities against different viruses. For example, PG1 showed antiviral activities against human immunodeficiency virus (HIV) [37], while MAP30 showed antiviral activity against herpes simplex virus (HSV) [38], HIV [39] and Kaposi's sarcoma-associated virus [40]. Based on our knowledge, there are no available studies illustrating the antiviral potential of PLSN except against dengue virus [16]. PG1 and PLSN were selected in this study based on their known inhibition potential against dengue NS2B-NS3pro. However, the results of viral binding assay showed that these peptides as well as the peptide-fusion protein also exhibited considerable inhibition against virus binding to the host cells. It could be possible that PG1 and PLSN individually or in fusion form may also act like some other cationic peptides such as retrocyclin which is effective in blocking viral attachment to host cell through its binding ability to heparan sulfate on the cell membrane [41], [42]. In parallel, the antiviral activity of MAP30 may depend on its ability in inhibiting host cells protein machinery (such as that shown by RIP) [23] and modulating some cellular genes necessary for viral and cell proliferation and apoptosis [40]. It is important to examine whether or not the antiviral activity of the peptide-fusion protein depends on the effects of MAP30 on cell growth and apoptosis. The results of real time proliferation assay showed that the peptide-fusion protein inhibited virus replication in infected cells with minimal effects on cell proliferation. These data are helpful to conclude that the antiviral activity of the peptide-fusion protein was independent from the possible effects of MAP30 on cell proliferation and apoptosis that have been reported by other studies [43], [44].

Based on the findings of this study, we assume that the peptide-fusion protein could potentially interrupt dengue life cycle. The considerable inhibition was observed at the binding stage and post-translational processes of the viral ployprotein, as evidenced by the inhibition of dengue NS2B-NS3pro. Such inhibition of viral protease could hinder flaviviruses replication and virion assembly, as evidenced by the lack of production of infectious virions in mutants carrying inactivating viral proteases [45]. However, other potentials such as the interruption of virus assembly and release from the infected cells need further investigations. Because the peptide-fusion protein consists of three different peptides, its mechanism of antiviral activity still requires additional examinations. Intriguingly, our study showed an evidence of potential efficacy of the peptide-fusion protein against viral propagation in mice challenged with lethal dose of DENV2. The results of the animal experiments demonstrate that the peptide-fusion protein exhibited significant antiviral efficacy against DENV2 propagation in the mice when the drug was intraperitoneally administered. These encouraging results warrant a need to further examine the efficacy of the peptide-fusion protein against dengue serotypes in sub-human primates, in which antiviral efficacy might be expected owing to the outstanding similarities with human in sites of virus replication.

In conclusion, individually MAP30, PG1 and PLSN showed considerable inhibition potential against dengue NS2B-NS3pro and dengue replication in cell culture. This activity was synergistically amplified in the recombinant peptide-fusion PG1-MAP30-PLSN protein. Our works suggests that identifying novel peptide-fusion protein with anti-dengue activity could lead to improved prevention and/or treatment strategies towards dengue infection. This finding could pave the way to the successful development of therapeutic anti-dengue agents.

## Supporting Information

Data S1 **Peptide fusion protein and DNA sequences and oligos design and characteristics that were used for DNA construction.**
(DOCX)

Table S1 **Design and characteristics of the oligos that were used to construct the peptide-fusion protein (PG1-MAP30-PLSN).**
(DOCX)
