# Supplementary material for: Fusion of Protegrin-1 and Plectasin to MAP30 Shows Significant Inhibition Activity against Dengue Virus Replication
Source: PLoS One. 2014 Apr 10;9(4):e94561. doi: 10.1371/journal.pone.0094561 (PMC3983197; doi:10.1371/journal.pone.0094561)
Supplement: Data S1 — Peptide fusion protein and DNA sequences and oligos design and characteristics that were used for DNA construction. (DOCX) [file pone.0094561.s001.docx]

**Supporting file**

**Peptide fusion protein and DNA sequences and oligos design and characteristics that were used for DNA construction.**

**(A)Protein Sequence**

RGGRLCYCRRRFCVCVGRVPGVGVPGVGDVNFDLSTATAKTYTKFIEDFRATLPFSHKVY
DIPLLYSTISDSRRFILLNLTSYAYETISVAIDVTNVYVVAYRTRDVSYFFKESPPEAYN
ILFKGTRKITLPYTGNYENLQTAAHKIRENIDLGLPALSSAITTLFYYNAQSAPSALLVL
IQTTAEAARFKYIERHVAKYVATNFKPNLAIISLENQWSALSKQIFLAQNQGGKFRNPVD
LIKPTGERFQVTNVDSDVVKGNIKLLLNSRASTADENFITTMTLLGESVVNVPGVGVPGV
GGFGCNGPWDEDDMQCHNHCKSIKGYKGGYCAKGGFVCKCY

**(B) DNA sequence optimized to E. coli preferred codon**

CGTGGTGGTCGTCTGTGCTACTGCCGTCGTCGTTTCTGCGTTTGCGTTGGTCGTGTTCCG
GGTGTTGGTGTTCCGGGTGTTGGTGACGTTAACTTCGACCTGTCTACCGCGACCGCGAAA
ACCTACACCAAATTCATCGAAGACTTCCGTGCGACCCTGCCGTTCTCTCACAAAGTTTAC
GACATCCCGCTGCTGTACTCTACCATCTCTGACTCTCGTCGTTTCATCCTGCTGAACCTG
ACCTCTTACGCGTACGAAACCATCTCTGTTGCGATCGACGTTACCAACGTTTACGTTGTT
GCGTACCGTACCCGTGACGTTTCTTACTTCTTCAAAGAATCTCCGCCGGAAGCGTACAAC
ATCCTGTTCAAAGGTACCCGTAAAATCACCCTGCCGTACACCGGTAACTACGAAAACCTG
CAGACCGCGGCGCACAAAATCCGTGAAAACATCGACCTGGGTCTGCCGGCGCTGTCTTCT
GCGATCACCACCCTGTTCTACTACAACGCGCAGTCTGCGCCGTCTGCGCTGCTGGTTCTG
ATCCAGACCACCGCGGAAGCGGCGCGTTTCAAATACATCGAACGTCACGTTGCGAAATAC
GTTGCGACCAACTTCAAACCGAACCTGGCGATCATCTCTCTGGAAAACCAGTGGTCTGCG
CTGTCTAAACAGATCTTCCTGGCGCAGAACCAGGGTGGTAAATTCCGTAACCCGGTTGAC
CTGATCAAACCGACCGGTGAACGTTTCCAGGTTACCAACGTTGACTCTGACGTTGTTAAA
GGTAACATCAAACTGCTGCTGAACTCTCGTGCGTCTACCGCGGACGAAAACTTCATCACC
ACCATGACCCTGCTGGGTGAATCTGTTGTTAACGTTCCGGGTGTTGGTGTTCCGGGTGTT
GGTGGTTTCGGTTGCAACGGTCCGTGGGACGAAGACGACATGCAGTGCCACAACCACTGC
AAATCTATCAAAGGTTACAAAGGTGGTTACTGCGCGAAAGGTGGTTTCGTTTGCAAATGC
TAC

**(C) Oligos design for DNA construction**

**Num Length Start-Stop Strand %G+C Tm Sequence (5'->3')**

1 60 1-60 + 60.0 78.3 CGTGGTGGTCGTCTGTGCTACTGCCGTCGTCGTTTCTGCGTTTGCGTTGGTCGTGTTCCG

2 60 46-105 - 55.0 76.2 AGACAGGTCGAAGTTAACGTCACCAACACCCGGAACACCAACACCCGGAACACGACCAAC

3 60 91-150 + 50.0 74.2 AACTTCGACCTGTCTACCGCGACCGCGAAAACCTACACCAAATTCATCGAAGACTTCCGT

4 60 136-195 - 55.0 76.2 CAGCAGCGGGATGTCGTAAACTTTGTGAGAGAACGGCAGGGTCGCACGGAAGTCTTCGAT

5 60 181-240 + 53.3 75.6 GACATCCCGCTGCTGTACTCTACCATCTCTGACTCTCGTCGTTTCATCCTGCTGAACCTG

6 60 226-285 - 51.7 74.9 GGTAACGTCGATCGCAACAGAGATGGTTTCGTACGCGTAAGAGGTCAGGTTCAGCAGGAT

7 60 271-330 + 50.0 74.2 GCGATCGACGTTACCAACGTTTACGTTGTTGCGTACCGTACCCGTGACGTTTCTTACTTC

8 60 316-375 - 45.0 72.1 ACCTTTGAACAGGATGTTGTACGCTTCCGGCGGAGATTCTTTGAAGAAGTAAGAAACGTC

9 60 361-420 + 48.3 73.5 ATCCTGTTCAAAGGTACCCGTAAAATCACCCTGCCGTACACCGGTAACTACGAAAACCTG

10 60 406-465 - 53.3 75.6 CAGACCCAGGTCGATGTTTTCACGGATTTTGTGCGCCGCGGTCTGCAGGTTTTCGTAGTT

11 60 451-510 + 60.0 78.3 ATCGACCTGGGTCTGCCGGCGCTGTCTTCTGCGATCACCACCCTGTTCTACTACAACGCG

12 60 496-555 - 61.7 79.0 CGCGGTGGTCTGGATCAGAACCAGCAGCGCAGACGGCGCAGACTGCGCGTTGTAGTAGAA

13 60 541-600 + 53.3 75.6 ATCCAGACCACCGCGGAAGCGGCGCGTTTCAAATACATCGAACGTCACGTTGCGAAATAC

14 60 586-645 - 50.0 74.2 TTCCAGAGAGATGATCGCCAGGTTCGGTTTGAAGTTGGTCGCAACGTATTTCGCAACGTG

15 60 631-690 + 50.0 74.2 ATCATCTCTCTGGAAAACCAGTGGTCTGCGCTGTCTAAACAGATCTTCCTGGCGCAGAAC

16 60 676-735 - 55.0 76.2 GGTCGGTTTGATCAGGTCAACCGGGTTACGGAATTTACCACCCTGGTTCTGCGCCAGGAA

17 60 721-780 + 46.7 72.8 CTGATCAAACCGACCGGTGAACGTTTCCAGGTTACCAACGTTGACTCTGACGTTGTTAAA

18 60 766-825 - 50.0 74.2 GTCCGCGGTAGACGCACGAGAGTTCAGCAGCAGTTTGATGTTACCTTTAACAACGTCAGA

19 60 811-870 + 53.3 75.6 GCGTCTACCGCGGACGAAAACTTCATCACCACCATGACCCTGCTGGGTGAATCTGTTGTT

20 60 856-915 - 53.3 75.6 GCAACCGAAACCACCAACACCCGGAACACCAACACCCGGAACGTTAACAACAGATTCACC

21 60 901-960 + 60.0 78.3 GGTGGTTTCGGTTGCAACGGTCCGTGGGACGAAGACGACATGCAGTGCCACAACCACTGC

22 60 946-1005 - 46.7 72.8 ACCACCTTTCGCGCAGTAACCACCTTTGTAACCTTTGATAGATTTGCAGTGGTTGTGGCA

23 58 966-1025 + 43.1 71.0 TATCAAAGGTTACAAAGGTGGTTACTGCGCGAAAGGTGGTTTCGTTTGCAAATGCTAC
